# Antibiotic sensitivity profile of bacterial isolates from stool samples among children below five years in Murang'a County, Kenya

**DOI:** 10.11604/pamj.2023.45.87.17909

**Published:** 2023-06-20

**Authors:** Oliver Waithaka Mbuthia, Musa Otieno Ng'ayo

**Affiliations:** 1 Department of Medical Microbiology and Immunology, School of Medicine, Faculty of Health Sciences, University of Nairobi, Nairobi, Kenya,; 2 Kenya Medical Research Institute, Center for Microbiology Research, Nairobi, Kenya

**Keywords:** Diarrhea, antibiotics, bacterial resistance, antibiotic resistance, mutation

## Abstract

**Introduction:**

the discovery of antibiotics led to the optimistic belief of completely eradicating infectious diseases during the golden era following their discovery. Countries are grappling with the burden of microbial resistance bringing a near paralysis of all facets of mankind. Enterobacteriaceae and other hard-to-treat Gram-negative bacteria have become resistant to nearly all antibiotic options available, and this is a bad taste in the fight against microbial resistance.

**Methods:**

during the months of April-October 2017, 163 children below five years presenting with diarrhea were randomly selected in Murang´a and Muriranja´s Hospitals. Bacterial agents were identified and antibiotic susceptibility profile was determined. Design: a cross-sectional study approach was used. Statistical analyses were performed using STATA v. 13.

**Results:**

a total of 188 bacteria belonging to 11 genera were isolated, and identified and their antibiotic susceptibility profiles were determined. Susceptibility testing showed that almost all the Enterotoxigenic Escherichia coli (ETEC), Enteropathogenic Escherichia coli (EPEC), Enteroaggregative Escherichia coli (EAEC), Salmonella, Klebsiella, Shigella, Vibrio, Enterobacter, Proteus, Pseudomonas, Aeromonas, Citrobacter and Yersinia species were resistant to the following antibiotics: ampicillin, amoxicillin, chloramphenicol, ciprofloxacin, ceftriaxone and kanamycin. Other than ETEC (90.9%), all the rest of the isolates were resistant to nalidixic acid. Other than ETEC (9.1%), EAEC (33.3%) and Salmonella species (95.2%), all the rest of the isolates were resistant to gentamicin. Other than V. cholerae, all the other isolates were resistant to trimethoprim-sulfamethoxazole. Isolates were sporadically resistant to erythromycin, streptomycin, doxycycline, and ofloxacin.

**Conclusion:**

the high resistance rate of enteric Gram-negative bacterial pathogens in Murang´a County is alarming. The need for urgent, efficient, and sustainable actions and interventions, such as culture and susceptibility testing, is needed and must be taken into account to minimize and prevent the establishment and spread of enteric pathogenic bacteria.

## Introduction

The widespread microbial resistance has been blamed for the complexity of today's bacterial infection management and therapy. It also places a significant economic burden on any Nation. It is predicted that by 2050, microbial resistance will cost over $100 trillion and cause over 10 million fatalities compared to the present low estimate of ~700,000 persons [1]. Antibiotic resistance underpins major advances in the treatment and management of infectious diseases. Undoubtedly, the emergence and dissemination of resistant bacterial strains, which is today`s major global public health threat, jeopardizes the efforts gained over the years [2]. Indeed, antibiotic resistance has impacted all facets of mankind, causing immense human suffering, extended hospital admissions, expensive healthcare access, and a significant number of deaths [3].

The overuse of limited antibiotic choices compounded by lagging in the discovery of vaccines and alternatives induce bacterial diversity and the evolution of bacterial resistance. Some bacterial illnesses can no longer be treated with carbapenems, which are advised as a “treatment of last resort” [4]. In addition, some microbes, such as Carbapenem-resistant *Enterobacteriaceae* (CRE), have diminished confidence in their effectiveness, and the variety of available antibiotics has decreased [5]. While food-borne and poverty-related attributes are additional drivers of bacterial resistance acquisition [6], mobile genetic elements across and/or among similar or different bacterial species [7,8], or by spontaneous mutations seriously jeopardize the efficacy of antibiotics [9]. Antibiotic overuse in agriculture has also invited resistance via the food chain and often comparable chemical compounds are now more frequently employed in the production of human antibiotics. In sub-Saharan Africa, microbial resistance to antibiotics is generally rising, and it is uncertain how quickly the issue is spreading [10].

Injudicious antibiotics use is most responsible for their ineffectiveness [1]. It gets worse because community setups, with little to no surveillance data, commonly use empirical antibiotic prescriptions without subjecting isolates to a variety of antibiotics. Stool samples are rarely investigated for bacterial pathogens among children below five years in most parts of Kenya which can be attributed to a lack and/or inadequate diagnostic capacity [10] and the absence of tracking systems to monitor antibiotic resistance. The limited diagnostic tools and technologies, particularly in developing countries, raise questions about the dedication of nations to the fight against antibiotic resistance. Development of vaccines and alternatives, sanitation and hygiene offer long-term solutions but an urgent step-up from the slow technology of diagnostic tools should be seen as an urgent remedy for real-time detection of bacterial resistance and at the same time optimize treatment and halt the spread that is already present. Bacterial resistance is a multifaceted issue that requires combined efforts [1], and indeed requires promoting awareness of antibiotic stewardship and advancing sustainable innovations targeted at preserving and discovering effective antibiotics.

## Methods

**Study site:** the study was conducted in Kenya's Murang'a County, which is around 80 kilometres from Nairobi, the country's capital. The selected public hospitals were based on their geographical location, either rural or urban. Murang´a Level (V) Hospital is the county’s largest referral hospital offering specialized diagnostic, curative, surgical, preventive and rehabilitative medical services. It hosts outpatient and inpatient departments. Muriranja’s Level (IV) Hospital on the other hand is situated approximately 100 kilometres from Nairobi. The hospital offers basic to specialized outpatient and inpatient medical services to the rural residents of Kiharu sub-County and the neighboring sub-Counties.

**Research design:** the study utilized a cross-sectional study design.

**Target population:** the study examined young children under the age of five who visited Murang'a Level (V) Hospital and Muriranja's Level (IV) Hospital for the treatment of diarrheal sickness. The child's carer provided written informed consent before a stool sample was taken.

**Inclusion criteria:** children below five years who reported a loose stool at Murang’a Level (V) Hospital and/or Muriranja’s Level (IV) Hospital, were residents of Murang’a County and caretakers of those who gave informed consent to participate in the study.

**Sampling design:** sample selection was done using systematic random sampling, whereby, the first unit (case) was selected randomly in each hospital. The n^th^ case after the starting point followed a systematic selection. The n^th^ case represents the sampling interval which was calculated by dividing the approximate total number of diarrhea cases by the sample size of 163 per facility. Therefore, every 4^th^ case of diarrhea in Muriranja’s Level (IV) Hospital and 5^th^ in Murang’a Level (V) Hospital were selected until a sample size of 163 was reached from both hospitals.

**Sample size determination:** using Daniel *et al*. [11] formula for estimating population proportion with specified relative precision and a detection rate of 12.1% for children under five who were diarrheal disease-infected in Murang'a County [12], a total of 163 children were recruited to achieve 0.95 power.

**Data collection instruments:** the official laboratory request form included the patient`s demographic, clinical information, and a presumptive diagnosis filled in the attending clinician. Individual patients´ names were dropped and a unique identification number that matched the stool sample label, questionnaire and a tailored study guide form was used. Alongside the detailed questionnaire, a study guide tool was developed to capture and fill in essential laboratory data. A section of the study guide form contained information on stool physical appearance, methods used to identify bacteria, taxonomic classification, species and strain identification, generic names of employed antibiotics, and their minimum inhibitory concentration.

**Validity:** pre-testing was conducted in the two hospitals before validating the research methods and tools. Sample storage, preservation, and transportation protocol were strictly followed per the institutional guidelines. Standardized operating procedures were used for all laboratory studies, setting standards and controls (positive and negative) wherever necessary. All the reagents were prepared in accordance with the manufacturers' instructions.

**Sample collection:** registered clinicians physically and clinically examined the child`s general health status and recorded it. Research assistants guided the child`s guardian on the questions contained in the questionnaire and ensured that a stool sample was correctly obtained. Diarrheal stool samples were collected on the day of presentation at the hospitals using well-labelled sterile leak-proof polypots. Stool appearance was recorded in the hospital laboratory study guide and on the study questionnaire entries that matched the specimen identification number. Stool samples were cultured in Cary Blair medium (Oxoid, United Kingdom) which were then properly sealed, labelled, and stored at 4-8 °C for one day. Samples were disposed of as per the standard operating procedure for infectious material. On the second day, the cultured transport media was put in a cool box with ice packs and transported within 3 hours to the Kenya Medical Research Institute (KEMRI), Center for Microbiology Research in Nairobi.

**Laboratory analysis:** using the appropriate media, all stool samples were instantly cultivated. From our previously published work, the specific steps, stool culture techniques used, bacteria isolates acquired, and their identification from stool samples of the study participants are discussed [13]. Furthermore, variables connected to the isolated enteric Gram-negative pathogenic bacteria were also examined [14]. Isolates were tested for their antibiotic susceptibility by the Kirby-Bauer disk diffusion method following the Clinical and Laboratory Standards Institute (CLSI) protocol [15].

**Data analysis and presentation:** raw data were entered, cleaned, edited, coded, and analyzed using STATA v 13 (StataCorp LP, College Station, TX, USA). Frequency (%), mean, standard deviation and medium (interquartile ranges at 25% and 27%) were used to describe the qualitative and laboratory parameters. Categorical variables were analyzed and interpreted using descriptive statistics. This data was presented on frequency tables and then described per the test characteristics (resistance, intermediate, sensitive).

**Ethical consideration:** ethical approval was granted by Kenyatta University Ethics and Research Review Committee; Ref No: KU/ERC/APPROVAL/VOL.1 (31). A research permit was given by the National Commission for Science, Technology, and Innovation; Ref No: NACOSTI/P/17/15949/16819. Additional approval was given by the County Commissioner of Murang´a County and the Directors of the Ministries of Health and Education in Murang´a County. The study was performed per the Helsinki Declaration [16]. Ethical standards including the protection and rights of participants were adhered to and signed consent was ensured before the commencement of the participant recruitment.

## Results

Table 1 describes the antibiotic susceptibility profile of enteric pathogenic bacteria isolated from the study participants. Isolates were tested for their antibiotic susceptibility by the Kirby-Bauer disk diffusion method according to Clinical and Laboratory Standards Institute (CLSI) guidelines. The drugs tested included: ampicillin (AMP), amoxicillin (AMO), chloramphenicol (CHLO), doxycycline (DOX), streptomycin (STR), tetracycline (TET), ciprofloxacin (CIP), nalidixic acid (NA), gentamicin (GEN), trimethoprim-sulfamethoxazole (SXT), erythromycin (ERY), ofloxacin (OF), ceftriaxone (CE), and kanamycin (K). The antimicrobial susceptibility was classified using the CLSI guidelines, as susceptible, intermediate, or resistant to each antibiotic. In addition, we also classified the isolates as either non-susceptible (including both intermediate and resistant isolates) or susceptible.

**Table 1:** distribution in antibiotic susceptibility profile by enteric pathogenic bacteria isolated from the study population

Isolates	Total	Antibiotics													
		**AMP**	**AMO**	**CHLO**	**DOX**	**ERY**	**GEN**	**NA**	**OF**	**STR**	**TET**	**SXT**	**CE**	**CIP**	**K**
*ETEC*	8	8(100)	8(100)	8(100)	8(81.8)	1(9.1)	1(9.1)	10(90.9)	10(90.9)	1(9.1)	10(90.9)	8(100)	8(100)	8(100)	8(100)
*EPEC*	3	3(100)	3(100)	3(100)	3(100)	0	3(100)	3(100)	3(100)	1(33.3)	2(67.7)	3(100)	3(100)	3(100)	3(100)
*EAEC*	2	2(100)	2(100)	2(100)	1(50)	1(50)	1(50)	2(100)	1(50)	1(50)	1(50)	2(100)	2(100)	2(100)	2(100)
*Salmonella spp*	21	21(100)	21(100)	21(100)	21(100)	1(4.8)	20(95.2)	21(100)	21(100)	2(9.5)	5(23.8)	20(95.2)	21(100)	21(100)	21(100)
*Klebsiella spp*	12	12(100)	12(100)	12(100)	10(83.3)	1(8.3)	12(100)	12(100)	12(100)	1(8.3)	9(75)	12(100)	12(100)	12(100)	12(100)
*Shigella spp*	14	14(100)	14(100)	14(100)	12(85.7)	1(7.1)	14(100)	14(100)	14(100)	0	12(85.7)	14(100)	14(100)	14(100)	14(100)
*Vibrio cholera*	1	1(100)	1(100)	1(100)	0	0	1(100)	1(100)	0	1(100)	1(100)	0	1(100)	1(100)	1(100)
*Enterobacter spp*	7	7(100)	7(100)	7(100)	4(57.1)	1(14.3)	7(100)	7(100)	7(100)	1(14.3)	4(57.1)	7(100)	7(100)	7(100)	7(100)
*Proteus species*	8	8(100)	8(100)	8(100)	5(62.5)	0	8(100)	8(100)	8(100)	0	7&87.5)	8(100)	8(100)	8(100)	8(100)
*Pseudomonas spp*	14	14(100)	14(100)	14(100)	12(85.7)	1(7.1)	14(100)	14(100)	14(100)	3(21.4)	6(42.9)	14(100)	14(100)	14(100)	14(100)
*Aeromonas spp*	8	8(100)	8(100)	8(100)	8(100)	0	8(100)	8(100)	8(100)	1(12.5)	8(100)	8(100)	8(100)	8(100)	8(100)
*Citrobacter spp*	3	3(100)	3(100)	3(100)	1(33.3)	0	3(100)	3(100)	0	3(100)	3(100)	2(66)	3(100)	3(100)	3(100)
*Yersinia enterocolitica*	2	2(100)	2(100)	2(100)	1(5)	0	2(100)	2(100)	2(100)	0	0	2(100)	2(100)	2(100)	2(100)

ETEC: enterotoxigenic *E. coli*; EPEC: enteropathogenic *E. coli*; EAEC: enteroaggregative *E. coli*; AMP: ampicillin; AMO: amoxicillin; CHLO: chloramphenicol; DOX: doxycycline; STR: streptomycin; TET: tetracycline; CIP: ciprofloxacin; NA: nalidixic acid; GEN: gentamicin; SXT: trimethoprim-sulfamethoxazole; ERY: erythromycin; OF: ofloxacin; CE: ceftriaxone; K: kanamycin; spp-species

Susceptibility testing showed that almost all the ETEC, EPEC, EAEC, *Salmonella, Klebsiella, Shigella, Vibrio, Enterobacter, Proteus, Pseudomonas, Aeromonas, Citrobacter* and *Yersinia* species were resistant to the following antibiotics: ampicillin, amoxicillin, chloramphenicol, ciprofloxacin, ceftriaxone, and kanamycin. Other than ETEC (90.9%), all the rest of the isolates were resistant to nalidixic acid. Other than ETEC (9.1%), EAEC (33.3%) and *Salmonella* species (95.2%), all the rest of the isolates were resistant to gentamicin. Other than *Vibrio cholerae*, all the other isolates were resistant to trimethoprim-sulfamethoxazole. Isolates were sporadically resistant to erythromycin, streptomycin, doxycycline, and ofloxacin (Table 1).

## Discussion

**Distribution in antibiotic susceptibility profile by enteric pathogenic bacteria isolated from the study population:** a high resistance rate of the isolates was noted on quinolones (OF, CIP, NAL) and this finding runs consistent with previous studies in Kenya [17]. Further, almost all isolates were highly resistant to cephalosporin such as ceftriaxone (CEF), aminoglycosides such as gentamycin (GEN) and kanamycin (K), penicillin (AMP, AMOX), sulfonamides (SXT) as well as chloramphenicol. These drugs are often used in the treatment and management of diarrheal illnesses associated with bacterial infections. The present study paints a clear picture of medical practitioners in the study region running out of antibiotic alternatives. The resistance of *Enterobacteriaceae* strains to even newer versions of third-generation cephalosporin has been reported in different regions [5]. Surprisingly, carbapenems-resistant *Enterobacteriaceae* has been reported [5]. Multi-drug resistant (MDR) bacterial pathogens are a global challenge [2] and have previously been reported in Kenya [18] and elsewhere [19].

The findings of this study are generally compatible with those of Qu *et al*. [20] in China who described a high resistant rate of *Shigella* on similar drugs, although our finding that STR was 100% sensitive differed sharply from the Chinese study that documented 100% resistance of *Shigella* to STR. It was also noted that *Shigella* achieved a nearly 93% sensitivity rate when subjected to ERY, contrary to the finding from Mulatu *et al*. [19]. *Shigella* species was 85.7% resistant to TET which can be compared to the finding from Sang *et al*. [17] who documented that *Shigella* resistance ranged from 50-100% on the same antibiotic. Pathogenic *E. coli* in this study was 100% resistant to SXT, AMP, and TET and this trend was observed in a study across 4 provinces in Kenya that reported a high resistance rate of pathogenic *E. coli* when subjected to similar drugs [17]. In line with the finding in this study, Boru *et al*. [21] found that all stool isolates from the Eastleigh urban refugee children were resistant to amoxicillin and closely comparable to Karambu *et al*. [22] who reported a 95% resistance rate of isolates to AMO. It is worth noting that all intermediate results on antibiotic sensitivity in our study were regarded as ineffective in the treatment of associated diarrhea illness.

*Vibrio* species subtype O1 was isolated from a stool sample and showed 100% sensitivity to DOX, ERY, OF and trimethoprim-sulfamethoxazole (STX) but 100% resistance to TET unlike what was observed by Boru *et al*. [21] who showed the sensitivity of this subtype to TET. The pathogen was also resistant to chloramphenicol (CHLO) and NAL similar to what was reported by Sang *et al*. [17] although our findings differed on its sensitivity to ciprofloxacin (CIP), GEN, and TET which was previously reported sensitive in some regions of Kenya. In most instances, *V. cholera* is associated with poor sanitation resulting in outbreaks [23]. It may be that *Vibrio* species is rare among the study population and such drugs are therefore not often used which limits bacterial resistance. In reality, at the time of the study, the isolated *V. cholera* may have been linked to the ongoing cholera nationwide outbreak. In most regions, however, CIP has been reported as sensitive to most *Enterobacteriaceae* [24]. It should be noted that CIP should be used cautiously because of its association with musculoskeletal toxicity although current studies have now recommended its use among neonates [25]. It is necessary to conduct additional high-quality clinical trials to assess its efficacy and safety in addition to its pharmacokinetics. Shigellosis pathogenic *E. coli* infection and salmonellosis appeared to be more difficult to treat because the associated pathogens are common in the study area [13] and, therefore, have a higher chance of injudicious exposure to the most commonly used antibiotics.

Empirical prescription of broad-spectrum antibiotics during non-bacterial infections or even when the diagnosis is unknown is rampant [26]. This behaviour has contributed immensely to antibiotic resistance posing a public health threat in many parts of the world [2]. For instance, medics use their expertise, intuition and professional judgment to “guess” the presence of a bacterial infection, which may lead to empirical antibiotic prescription before the use of diagnostic tools to confirm the diagnosis. In Western Kenya, out of 112 children prescribed empirically with antibiotics in Asembo, 51% of isolates were later found to be resistant, and 84% of those infected with *Shigella* had been prescribed resistant antibiotics [27]. This study is an interesting example demonstrating proof evidence of injudicious prescription of antibiotics often abnegating the appropriate laboratory diagnostic aspect. This problem is amplified by the inability of primary healthcare facilities to subject bacterial isolates to an array of antibiotics to assess their susceptibility, especially, within the study area. The phenomenon of prescribing antibiotics empirically has been documented to be strongly associated with high microbial resistance in many studies [28].

Another possible reason could be the diversity of virulence genes harboured by bacterial isolates within the study region [25]. Data on bacterial genes conferring resistance to the commonly used antibiotics in the study area was limited. However, findings from a study in Brazil showed that almost all *Shigella species* isolates from infant stool samples possessed *ipa* virulence genes evading HeLa cells which alerted MDR-shigella [29]. Probably, bacterial virulence genes and their diversity could be the hallmark of the observed high rates of bacterial resistance to antibiotics within the study region. From the current study, pathogenic *E. coli* carried virulence genes that may have contributed to bacterial resistance when subjected to various antibiotics. These findings call for the need to conduct studies that evaluate different gene mutations conferring antibiotic resistance, which otherwise, would provide insights into the future development of novel diagnostic, therapeutic, and preventive tools. Likewise, gene transfer to recipient bacterial etiologies has been shown to narrow the antibiotic arsenal. Conjugation experiments have demonstrated that gene transfer elements such as blaCTX-M, inducible AmpC beta-lactamase, and MOX genes were transmissible to the *E. coli* J53 strain in the presence of ceftriaxone [30]. The importance of bacterial isolation followed by antibiotic susceptibility testing remains paramount, especially, *Aeromonas* species whose MDR has been reported in Brazil [31] and Egypt [32] but showed a 100% sensitivity to ERY (100%) and 12.5% for STR in this study.

Plasmids harboured by MDR-*Enterobacteriaceae* remain an unexplored area despite studies indicating that plasmids are the hotspot for genetic spread rendering antibiotics ineffective. *Klebsiella, Enterobacter, Proteus, Pseudomonas, Citrobacter* and *Yersinia* species were resistant to AMP, AMO, CHLO, CIP, CEF and K. Conjugation of genetic elements such as a pCTX-M3 plasmid, responsible for the spread of extended-spectrum B-lactamase (ESBL), is easily transferred from *E. coli, Klebsiella* and *Enterobacter* species [33]. Some ESBL-producing *Enterobacteriaceae* have been documented to be resistant to almost all penicillin and cephalosporin classes giving leeway for carbapenem use [4]. The majority of these antibiotics are continuously prescribed for the treatment of diarrheal illness among children. Recent studies have tried to investigate asymptomatic reservoirs for bacterial strain and resistant gene dispersion. A study in Nigeria identified genetic elements from healthy mothers and their children who presented with diarrhea and reported mother-child pairs (10.3%), some showing identical genetic elements of diarrheagenic *E. coli* to antibiotic resistance mediated by classes of integrons acquired horizontally [34]. Bacterial reservoirs are a controversial topic that requires more investigation to close the loophole, whereby, these bacteria likely contribute to the resistance of other bacteria through gene transfer.

## Conclusion

Erythromycin and streptomycin had the minimum bacterial resistance among major pathogenic bacterial isolates associated with an enteric bacterial infection in Murang’a Level (V) and Muriranja’s Level (IV) Hospitals in Kenya. The continuous emergence and spread of bacterial strains greatly jeopardize antibiotic ability in the treatment of enteric Gram-negative bacterial agents causing diarrhea among children below five years in the study area. Action to improve local, national and global bacterial resistance surveillance systems to predict future trends complement immediate action to address the antimicrobial resistance crisis. The study recommends the need to empower and promote laboratory capacity to unfold the changing microbial resistance and prevent its emergence and spread.

### What is known about this topic

- *Inappropriate use of antibiotics is a leading cause of bacterial resistance;*
- *MDR microbes are a current global public health threat making treatment and management of infectious diseases difficult*.

### What this study adds

- *MDR-Enterobacteriaceae among children below five years is a crisis in Murang’a County and poses a public health emergency if ignored;*
- *Antibiotic sensitivity profiling of isolated Enterobacteriaceae from stool samples of children below 5 years provides data that can be used to develop antibiograms within the region*.
